# Co-designing interventions to ‘live well’: experiences and perceptions of the Genetic, Undiagnosed and Rare Disease (GUaRD) community

**DOI:** 10.1007/s12687-023-00643-1

**Published:** 2023-03-31

**Authors:** Inez Beadell, Malia Byun, Hollie Feller, Monica Ferrie, Stephanie Best

**Affiliations:** 1 Department of Medicine, Austin Health, University of Melbourne, Melbourne, Australia; 2 California Lutheran University, Thousand Oaks, CA USA; 3 Genetic Support Network Victoria, Melbourne, VIC Australia; 4 Department of Health Services Research, Peter MacCallum Cancer Centre, Parkville, Melbourne, Australia; 5 Victorian Comprehensive Cancer Centre Alliance, Parkville, Melbourne, Australia; 6 Australian Genomics, Murdoch Children’s Research Institute, Melbourne, Australia; 7 Sir Peter MacCallum Department of Oncology, University of Melbourne, Melbourne, Australia

**Keywords:** Intervention, ‘Live well’, Genetic, Undiagnosed and Rare Disease, APEASE, Qualitative, Co-design

## Abstract

The Genetic, Undiagnosed and Rare Disease community faces a range of hurdles to live their ‘best life’ including physical, social and psychological barriers. They are also resilient and experiential experts with insight into what works and what could work for them. In this study, we aimed to identify and prioritise practical interventions the Genetic, Undiagnosed and Rare Disease community report could help them to ‘live well’. Using a three-stage approach, we first analysed data from a year-long Genetic, Undiagnosed and Rare Disease journal study to record all the practical interventions reported, either trialled or proposed. Second, after grouping the interventions (*n* = 19) into four themes (support for individuals with GUaRD; support for carers of people with GUaRD; education/employment; transition), we presented the interventions to members of the GUaRD community (people with GUaRD, their carers and peer support group members) across three focus groups (*n* = 13). Focus group transcripts were analysed for refinements to the interventions, barriers and/or enablers to enacting them and for any additional interventions suggested. From this analysis, the interventions were grouped to identify specific actionable activities (*n* = 8). Finally, these eight interventions were discussed in a workshop with the GUaRD Community Advisory Group and prioritised using the APEASE framework. Prioritised interventions targeted a range of stakeholders and included creating a lived experience video library, supporting peer support groups with grant applications, and educating clinicians about referring to peer support groups. Further research is now required to test these findings before trialling and evaluating an intervention to measure the impact on the GUaRD community.

**Supplementary information:**

The online version contains supplementary material available at 10.1007/s12687-023-00643-1.

## Introduction

The Genetic, Undiagnosed and Rare Disease (GUaRD) community is a broad group of people with individually rare conditions that when considered collectively are relatively common. Three and a half percent to 5.9% of people worldwide (Nguengang Wakap et al. 2020) have either a genetic, undiagnosed or rare disease. While the symptoms people present with are varied, they are united by the chronic ongoing nature of their conditions (Depping et al. 2021; Uhlenbusch et al. 2019). Research frequently focuses on the barriers facing this community providing insights into their lived experience, often reporting on individual conditions (e.g. sarcoidosis (Moor et al. 2018)), and these studies provide valuable understanding of specific communities. However, this population faces a range of common challenges from getting a diagnosis, securing ongoing health and/or social care and provision/support for long-term carers (Anderson et al. 2013; Zurynski et al. 2017a, b). The GUaRD community is resilient and resourceful (Byun et al. 2022), and they are the experts in their condition (Budych et al. 2012). As such, they are ideally placed to identify enablers/solutions to improve the quality of life for many people in the community.

Visibility of people with genetic, undiagnosed or rare disease increased with the United Nations General Assembly resolution for people living with rare disease. This declaration acknowledged the need for equal access to a ‘standard of living adequate for the health and well-being of oneself and one’s family’ (United Nations 2021). To achieve this aim of ‘living well’, access to clinical services and support such as community services, community networks, patient support groups and family and peer support is critical to the wellbeing and mental health of newly diagnosed, non-diagnosed and those on their continuing personal care journey through the healthcare system while navigating the service sector. Positive change will require collaboration, with and across government, industry, researchers, health professionals, social services and patient support organisations and community (Alderwick et al. 2021; Dowling et al. 2004).

The impact of consumer engagement in research is well established and involves ‘research being carried out “with” or “by” members of the public rather than “to”, “about” or “for” them’ (INVOLVE The National Institute for Health Research 2019). Benefits of engaging with consumers have long been recognised (Arnstein 1969) and include, improved relevance of research to patient needs, improved quality and outcomes, improved public confidence in research, etc. (Todd and Nutbeam 2018). However, the emphasis is often on the challenge community face rather than what can go well.

Examples of taking a positive and proactive approach can be found in the literature, e.g. safety II where safety in healthcare is examined from the perspective of what goes right (Hollnagel et al. 2015), appreciative inquiry which provides a positive structured approach to examining organisational change (Cooperrider Barrett and Srivastva 1995) and Positive Organisational Scholarship in Healthcare (POSH) where the focus is on celebrating excellence (Dadich et al. 2015). These methods provide an empowering emphasis with which a community can identify what has succeeded and what they need more of. With the GUaRD community, we take a positive approach to examine what can contribute to living well by identifying practical interventions to ‘live well’. For this study, we deem ‘practical interventions’ to mean intentional actions designed to deliver a positive health or wellbeing outcome.

The aim of this co-designed and co-led study was to investigate the GUaRD community’s perceptions of practical interventions that could improve their quality of life. Study objectives were to (i) identify and (ii) prioritise practical interventions, the GUaRD community report could help them to ‘live well’.

## Material and methods

### Context

In Australia, current conservative estimates indicate that between 6 and 8% of the population is affected by a rare disease (Elliott and Zurynski 2015) which is comparable with the number of people with diabetes (Australian Bureau of Statistics, 2022). The Genetic Support Network Victoria (GSNV) (https://www.gsnv.org.au/), a statewide organisation focused on supporting people living with GUaRD and those who support them, and Australian Genomics (https://www.australiangenomics.org.au/), a research funded network supporting implementation of genomic medicine, collaborated to co-design and deliver the study.

### Study design

**Fig. 1:**
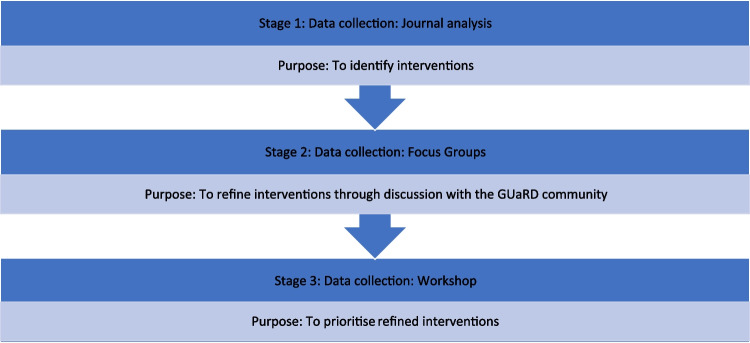
Study design by stage with data collection and purpose. Key: GUaRD, Genetic, Undiagnosed and Rare Disease

Recognising the importance of community perspectives in developing knowledge, we used a social constructivist approach (Hennick et al. 2020) employing a multi-staged qualitative design (Fig. 1). First, secondary data analysis was undertaken on journals from a year-long study to identify practical interventions. Second, focus groups were held with the GUaRD community to discuss the interventions. Finally, a workshop was held to prioritise the interventions.

#### Stage 1

Journals were collected from members of the GUaRD community from July 2020 to May 2021 to record their day-to-day experiences. This study is reported in full elsewhere (Byun et al. 2022), and in summary, 27 people took part, recruited via the GSNV social media. Many had several roles in the GUaRD community: 11 with a GUaRD, 7 were carers and 6 coordinated support groups. Participant ages ranged from 18 to 75 with seven aged 46–59, six people aged 18–29, three people aged 30–45 and one between 60 and 75 years. Each month participants were invited, by email from the GSNV, to submit a journal in any format (text, graphics, video). There was no commitment to submit each month, and over the study period, there was an average of ten submissions a month.

##### *Data analysis*

Two researchers (IB and MB) familiarised themselves with the journals: one researcher had been part of the original journal study research team (MB). Data analysis was in two steps. First, contextual topics were identified where participants reported the need for additional support. Second, practical interventions were identified that aligned with the contextual topics. These interventions were cleaned and categorised using a modified version of the PICO (Richardson et al. 1995): context, population, intervention, comparison, and outcome (CoPICO) (Lockwood et al. 2015), i.e. *context*, an explanation of the problem or suggested intervention from the participant journals; *population*, characteristics of the population who the interventions were designed for; *intervention*, the intervention and those responsible for providing required resources; *comparison*, possible alternatives to the intervention; and *outcome*, the effects of that intervention on the population and any additional stakeholders. Themes and interventions from this analysis informed stage 2 focus groups. Weekly research meetings were held (with SB and HF) to provide guidance and resolve any conflicts through discussion of the analysis and identification of challenging coding.

#### Stage 2

We invited people from the GUaRD community to participate in a focus group to discuss and refine the interventions identified in the journals. Recruitment occurred through GUaRD-associated social media and websites (e.g. GSNV) and re-contacting participants from the initial COVID-19 journal project by email. Participants were eligible if they were a person with a genetic, undiagnosed or rare disease, were a carer and/or were part of a GUaRD support group. Focus group questions were piloted with a member of the GUaRD community and wording refined as required. The sessions were held online with two female researchers, led by SB (a highly experienced qualitative researcher working with Australian Genomics) supported by IB (a student researcher and GSNV volunteer). Sessions were audio recorded—additional field notes were not taken—and ran for about 90 min. Participants had no previous relationship with the researchers and were aware of the reasons for undertaking the research from the participant information sheet. The purpose of the focus group was to (i) share the practical interventions that arose from the journals study in relation to day-to-day lives and engagement with the health system and (ii) gather the groups’ perspectives on refining the interventions that had the potential to have impact on people’s lives. We held three focus groups between August to September 2021 with only the researchers and participants in attendance until a consensus in views was achieved. See online resource 1 for focus group protocol. A summary of the session was shared with participants after the focus groups.

##### *Data analysis*

Focus group transcripts were fully transcribed and anonymised before analysis with the aid of NVivo. Additional field notes were not taken. Four members of the research team (IB, MB, HF, SB) independently reviewed one of the transcripts to identify the practical interventions either supported or proposed by participants. Findings were discussed at the fortnightly team meeting before all transcripts were coded (IB and MB) using thematic analysis (Braun and Clarke 2006). First, interventions discussed were assigned initial labels (i.e. codes) before related codes were grouped into categories as refined practical interventions (IB, MB, HF). Practical examples were identified from the focus group transcripts with sensemaking (Weick et al. 2005) through the research team’s lived experience.

#### Stage 3

We sought the opinions of the GUARD Collaborative Australia, Community Advisory Group to prioritise the interventions refined in stage 2. All 12 members were invited, by email (MF), to an online workshop in February 2022. Only the participants and researcher were present. The session was led by SB, ran for about an hour and was audio recorded. The purpose of the session was to prioritise the refined interventions. However, prioritisation can be challenging with different participants focusing on different themes. As such, we used a framework to ensure emphasis was placed on targeted areas. Using the APEASE framework (Michie et al. 2005), designed for use in behaviour change in health care, we asked participants to score each intervention by affordability, practicability, effectiveness, acceptability, side effects and ethical considerations. See online resource 2 for workshop protocol. A summary of the session was shared with participants after the workshop.

##### *Data analysis*

The APEASE scorings were summed for each intervention with the highest and lowest scoring domains noted. The focus group was transcribed and discussion related to the interventions identified (SB and IB)—no additional field notes were taken. Findings were discussed in the regular research meeting.

All procedures followed were in accordance with the ethical standards of the responsible committee on human experimentation (institutional and national) and with the Helsinki Declaration of 1975, as revised in 2000. Informed consent was obtained from all patients for being included in the study. Approval was granted by the Human Research Ethics Committee at the Royal Children’s Hospital, Melbourne (26/06/2021; HREC/77492/RCHM-2021).

## Results

### Stage 1: CoPICO results

Table 1 provides one example from the CoPICO analysis (see online resource 3). From the journal analysis, we identified fourteen contextual topics that participants reported as areas that would benefit from additional support. The topics included social connections, activities and hobbies with similar adults, accommodation for individuals within specialised activities, career and education development, support facilitating independence for GUaRD children, educational supports, online work and commitments, accessible exercise, support for carers, database of rare disease specialists, increased rare disease education for healthcare professionals, accessible telehealth, mental health and wellbeing support for individuals with GUaRD, awareness of peer support and linking with case workers and support workers. Most of the contextual topics (*n* = 12) centred on providing support to people with GUaRD, one for both people with GUaRD and their carers, and one for healthcare providers (e.g. general practitioners and health specialists).

**Table 1:** Example of CoPICO analysis with one contextual topic, including exemplar quotes

Contextual topic with exemplar quotes	Population	Intervention	Comparison	Outcome
**Social connection, activities and hobbies with similar people (e.g. friends and GUaRD community**) ‘This is about social interaction and getting to know each other, joining in the conversations” (ID 1, Carer/Support sector) “When your child has a rare syndrome, connecting with others who understand and know about the syndrome is important. Social media has made the world a lot smaller and we can easily find other families or individuals online to share stories and learn from each other. But face to face, real life interaction offers so much more’ (ID 1, Carer/Support sector) ‘I feel a level of isolation generally anyway, with not knowing anyone else with my condition or even what exactly it is’ (ID 3, GUaRD) ‘It’s so hard for my daughter to make friends when she has a chronic medical condition and spends a lot of time in hospital’ (ID 27, GUaRD/Carer/Support sector)	GUaRD community	What: arranging activities and social groups for the GUaRD community (and promoting balance) Who: peer support groups	Little to no social connections	GUaRD: meet other people, improved connections, socialisation, enjoyment, independence, gain skills, alleviate sense of being ‘alone’ Carers: reduced workload, peace of mind Program/organisation: spent time and resources

In total, 16 practical interventions were identified, e.g. collect and compile a list of organisations, companies or other professionals who can accommodate individuals with GUaRD in specialised activities and arrange accessible education and work training opportunities (see online resource 3 for additional detail). Each intervention had at least one identified provider who would be able to deliver that intervention. Peer support groups were the most common ideal provider, and additional providers included Technical And Further Education (TAFE) opportunities, educational institutions and healthcare professionals. Each contextual topic and intervention yielded its own unique outcome; some outcomes were gaining independence, employment, developing skills, occupying time, given a sense of purpose and reduced workload for carers.

### Stage 2: focus groups 

#### Focus group participants

In total, 17 responded to our invitation to participate in a focus group with 13 attending. No reasons were given for the four people who did not participate. Most participants had multiple roles in the GUaRD community: seven had a GUaRD, five were carers, and nine were part of the support sector. Participants’ support group categories included syndromes with intellectual disability, respiratory, metabolic, connective tissue, neurodevelopmental, neuromuscular, neurological and mitochondrial disease (https://www.gsnv.org.au/directory-of-genetic-conditions/). The number of focus group participants ranged from two to six. Two participants were aged 18–29, five were 30–45, three were 46–59, two were 60–75, and one was 75 + years. Table 2 shows participant connection to the GUaRD community.

**Table 2:**
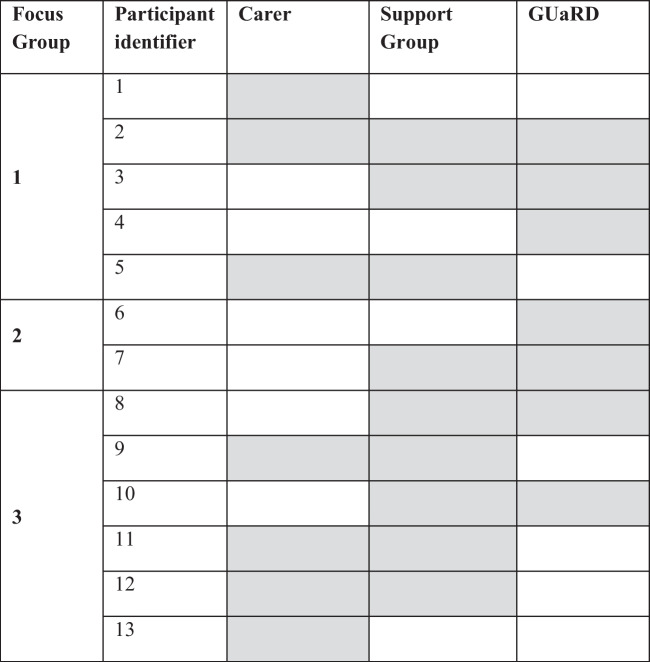
Focus group participant connection to the GUaRD community

Key: Shaded cells indicate participant connection to the GUaRD community; *GUaRD* Genetic Undiagnosed and Rare Disease

#### *Focus group result*s

We identified eight refined practical interventions (Table 3), for example, the use of creative approaches to generate social connection and creating information about the lived experience of having a GUaRD. Key stakeholders in these interventions included clinicians, peer support groups, people with GUaRD and carers either as an ideal provider of the intervention or the target/benefactor. Table 3 summarises these refined practical interventions using a title, descriptor and illustrative quotes from the focus groups.

**Table 3:** Summary of focus group refined practical interventions

Refined practical intervention	Descriptor	Ideal provider	Targeting	Illustrative quote
Clear referral process to credible peer support	Creating an accreditation system for peer support groups, then providing clinicians with a database of accredited peer support groups to refer to consumers	State health departments, local health organisations supported by support group peak bodies	People with a GUaRD, their families and carers	‘need…support groups for people because many of our people were feeling very isolated, very alone, had never met anyone like them before, but also…the support group provided them with information…and also provided them with lists of professionals…who were experienced with their condition’ (FG, ID 4)
Creativity in social connection	A breadth of inclusivity and accessibility in the connection of patients and their carers within the GUaRD community	Peer support groups	People with a GUaRD, their families and carers	‘we've been trying to set up face to face regional meetings as well, but the fact that they can just get online and join us has been fantastic. So the attendance by regional people and people who I know are really struggling to get around physically has been great’ (FG, ID 6)
Central rare disease portal/registry	A central portal/registry linked to the health system that contains key health information, including a list of support organisations for rare diseases in Australia	Federal health departments	People with a GUaRD, clinicians and researchers	‘I think information about the rare disease and the treatment guidelines have to be available 24/7 online’ (FG, ID 4)
Accessible mental health/wellbeing resources for clinics and homes	Addressing the overwhelming need for resources in the mental health area for the patient and their family with accessibility in hospitals/clinics and homes	Peer support peak bodies, supported by peak bodies	People with a GUaRD, their families and carers	‘we do need more mental health support and respite and…Getting support from education system…when the kids are at home and doing homework…or dealing with bullying and then bringing all that sort of stuff home with them and…making it really challenging in the home environment’ (FG, ID 8)
Supporting the supporters	Providing education, training and resources to patient support group organisations for the group and their carers	Peer support peak bodies	Peer support groups	‘the carer role is really challenging itself…and I think promoting the role and the needs of carers…could be enhanced so that there are carers actively and proactively seeking support…their mental health and well-being is just as important as for the patient’ (FG, ID 10)
Education about lived experience	Creating content that breaks down the GUaRD life journey to provide health professionals, educators and the community with detailed information about living with a rare disease	Peer support groups	General public, clinicians	‘I think helping people with rare conditions to effectively educate others would be a really good thing. How to better equip us to communicate with others about the limitations disease creates, but without doing it in a way that is threatening or is demeaning for us’ (FG, ID 6)
Transition support/education	Identifying the various stages of transition in a patient’s life journey and providing education to support these transitions	Peak clinician and health care bodies in conjunction with peer support groups	People with a GUaRD, their families and carers	‘If I had a good mentor on how to get a job with someone who likes someone who has a disability or acknowledges someone who has a disability, it would make more of a difference for me, I would be happier’ (FG, ID 1)
Peer-to-peer support	Providing mentor opportunities between members of the GUaRD community to support people emotionally, mentally and physically with their shared experiences	Peer support peak bodies; peer support groups	People with a GUaRD, their families and carers	‘when you’ve got a rare condition, having someone else, whether it’s other parents or another person who has the condition, it’s invaluable to have someone that you can connect with or talk about the challenges’ (FG, ID 5)

Key: *GUaRD*, Genetic, Undiagnosed and Rare Disease. Peak body is a non-government organisation whose membership consists of smaller organisations of allied interests. The peak body represents the interests of the members and offers a strong voice for the specific community sector in the areas of lobbying government, community education information sharing and other agreed objectives

Focus group participants reported that some of the interventions already existed yet were often inconsistent across support groups and locations.

### Stage 3: workshop

#### Workshop participants

In total, 9 participants attended the workshop with three not attending—no reasons were given for non-attendance. Some participants had multiple roles in the GUaRD community: one had a GUaRD, five were carers, and seven were part of the support sector. Two participants were aged 18–29, three were 30–45, and four were 46–59.

#### Workshop results

Participants used the APEASE framework to score examples of each intervention. Table 4 depicts the average score across the six APEASE criteria (affordability, practicability, effectiveness, acceptability, side effects and ethical considerations) for each example, with the highest and lowest criteria noted (full results can be found in online resource 4). The interventions are ranked from highest to lowest average score. The top scoring practical interventions were developing a *clear referral process to credible peer support*, though there was concern during the discussion over resistance from clinicians, and *peer-to-peer support* that were both perceived as being highly acceptable and affordable. The lowest scoring practical intervention was the generation of *central rare disease portal/registry* with concerns over potential effectiveness. One participant remarked, ‘what happens when people move on hospitals and you will get backlash for the extra work that clinicians have to do to enter it’ (WS Participant 7).

**Table 4:** APEASE results

Practical intervention	Example	Average score (0–10)	Highest score (0–10)	Lowest score (0–10)
Clear referral process to credible peer support	Educating clinicians about peer support groups	7.67	9.1, acceptability	6.6, affordability
Peer-to-peer support	Connecting people with common traits	7.67	9.2, acceptability	6.1, affordability
Transition support/education	Creating workshops that educate people with GUaRD and their families about transitioning into the workforce from school	7.22	8.7, effectiveness	5.8, equity
Accessible mental health/wellbeing resources for clinics and homes	Creating a database of mental health practitioners (e.g. psychologists) who are interested in rare disease and providing priority access to appointments	7.18	8.3, effectiveness	6.2, affordability
Education about lived experience	A lived experience video library	7.08	7.7, practicability	6.1, side effects/safety
Supporting the supporters	Support in grant writing to access funds for peer support group resources	7.02	8.3, acceptability	4.9, equity
Creativity in social connection	Creating face-to-face catch ups in each state or within groups of people with GUaRD who share common traits	6.63	7.7, effectiveness	4.7, equity
Central rare disease portal/registry	Adding summaries of rare diseases to existing government or hospital registries for health professionals and consumers	6.38	7.3, effectiveness	5.4, affordability

Key: *GUaRD*, Genetic, Undiagnosed and Rare Disease

## Discussion

This study has revealed and prioritised practical interventions to ‘live well’ as identified by people from the GUaRD community. The mainstay of the interventions was targeted directly at people with GUaRD across various time points in their lives, their carers, though the wider community including health professionals were also acknowledged, the latter having an indirect impact on the lives of people living with a GUaRD. Health professionals may be willing to support the GUaRD community though often need guidance to do so (Zurynski et al. 2017a, b). Delivery of the interventions was reported to be achieved through peer support groups, state government and/local health organisations—though a coordinated approach is likely to have the most value (Dowling et al. 2004).

On the whole, the emphasis was on provision of the interventions by peer support groups highlighting the role these groups can and do play. However, this prominence also raises issues of equity and access amongst the GUaRD community. GUaRD support groups vary in size from well established, funded and staffed groups such as Leukodystrophy Australia to smaller, volunteer run organisations such as Gorlin Syndrome Alliance. This difference means support groups have variable access to resources with smaller groups lacking time and extant resources to seek out more. Discrepancies also occur between groups depending on their stage of maturity. Greiner (1977) identifies key influences on the life cycle of organisations including age and size of the organisation, stages of evolution (i.e. growth), stages of revolution (i.e. turmoil) and growth rate of the industry (i.e. the market), several of which align to peer support groups (Srinivasan 2016). If the ‘growth rate of the industry’ (i.e. expectations of what peer support groups can provide) is disproportionate to evolution of the group, then there will be a misalignment of expectations which holds the potential to disappoint and frustrate both the GUaRD community and the peer support groups. Considered and proactive implementation is required to ensure peer support groups thrive (Fisher et al. 2015).

Mindful of competing demands on service providers, we drew on the APEASE framework to start to identify which interventions the community would prioritise to ensure maximise potential impact. For the top prioritised interventions (*clear referral process to credible peer support* and *peer-to-peer support*), the highest scoring APEASE domain was acceptability. Although not always a prerequisite to effectiveness, recipient perceptions of acceptability are central to the success of an intervention (Sekhon et al. 2017). Co-designing interventions with strong community involvement throughout delivery is essential to maximise resources and see support provided to the GUaRD community that meets their needs.

Interventions proposed included some that have been trialled, for example, within the intervention s*upporting the supporters*—peer support groups can be given assistance in grant writing to access funds for peer support resources. Generic examples exist (e.g. OurCommunity.com, a website that shares grants and provides basic grant writing tools; https://www.fundingcentre.com.au/); however, it is not bespoke to the GUaRD community. Tailored examples include regular sharing of available grant opportunities and support group bootcamps to share knowledge and tips about how to apply for grants (https://www.gsnv.org.au/community-professionals/support-groups/resources-for-support-groups/). Other interventions have not yet been implemented in Australia, e.g. developing a *central disease registry*. However, as part of the National Strategic Action Plan for Rare Diseases in Australia (Commonwealth of Australia 2019), federal funding has now been allocated to investigate and coordinate a national approach to rare disease registries (https://rarevoices.org.au/australian-rare-disease-registry-audit-project-update-august-to-october/).

While it is exciting to see support being provided to the GUaRD community, what is less clear is the impact and utility of these interventions for the community and whether they will bring about the outcomes intended. To optimise the impact of funding and maximise the outlay of resources, it is critical that interventions are trialled, with a rigorous evaluation, to assess what benefit they provide the GUaRD community (Lewis et al. 2015). This evaluation can determine whether an intervention should be expanded, mainstreamed or disinvested from. Next steps for this study include trialling the prioritised interventions to test whether they meet their intention of supporting people in the GUaRD community to live well.

This study has limitations. The variety of practical interventions may have been limited by accessing them during the SARS-CoV-2 pandemic; however, we mitigate this by undertaking focus groups to ensure we had a broad selection. We only had the opportunity to hold one prioritisation workshop, and although this was with an expert group, further prioritisation activities may be beneficial potentially including others noted in the practical interventions, e.g. clinicians. While there are advantages of using focus groups to collect data, e.g. interaction of participants stimulates discussion, there are also limitations which should be noted, e.g. some people may be unwilling to talk in a group. We did not capture a wide range of demographic information, e.g. socioeconomic status and racial/ethnic identity, and these factors could influence perspectives on both the interventions generated in stage 1, the revision in stage 2 and the prioritisation in stage 3. Future studies should ensure this data is collected and diverse population recruited. We used a framework in stage 3. Although the APEASE framework assists in structuring thinking, there may be other topics participants would prefer to use in order to prioritise interventions.

Drawing on the experiences and preferences of the GUaRD community, this study has identified and prioritised practical interventions that could promote ‘living well’. Using a multistage approach has revealed eight practical interventions that could be implemented to support the community. Preference was given to interventions perceived as more acceptable and affordable (*developing a clear referral process to credible peer support* and *peer-to-peer support*) while more complex interventions were prioritised lower (*developing a central disease registry*). It is essential that whichever practical interventions are implemented to support the GUaRD community to ‘live well’, they are evaluated to ensure people living with GUaRD gain maximum benefit. Central to this evaluation is the GUaRD community who should play a key role in the design and implementation of any activities aimed at impacting people with GUaRD.

## Supplementary information

Below is the link to the electronic supplementary material.

Supplementary file1 (DOCX 16 KB)

Supplementary file2 (DOCX 16 KB)

Supplementary file3 (DOCX 22 KB)

Supplementary file4 (DOCX 4252 KB)

## Declarations

### Disclaimer

The funders played no part in the study design; data collection, analysis, and interpretation; in the writing of the manuscript or in the decision to submit this manuscript for publication. The contents are solely the responsibility of the individual authors and do not reflect the views of the NHMRC.
