# Supplementary material for: Co-designing interventions to ‘live well’: experiences and perceptions of the Genetic, Undiagnosed and Rare Disease (GUaRD) community
Source: J Community Genet. 2023 Mar 31;14(3):295–305. doi: 10.1007/s12687-023-00643-1 (PMC10063929; doi:10.1007/s12687-023-00643-1)
Supplement: Supplementary file 1 — Supplementary file1 (DOCX 16 KB) [file 12687_2023_643_MOESM1_ESM.docx]

Supplementary file 1: Focus Group Protocol

**The Genetic, Undiagnosed and Rare Disease community: ‘Living well’**

**Welcome and establish ground rules for the focus group**

**Any questions before we get started?**

**Overview to the research plan**

Today we plan to:

1. Share practical interventions that arose from the journals study in relation to day to day lives and engagement with the health system

2. Get your thoughts on which ones may be helpful to improve people’s lives

Share themes with participants. Examples of discussion prompts include

Is this something you are familiar with already?

How does it work for you? OR how could this look in real life?

Could it be of value?

In what way?

For who?

By who?

How could it be improved OR provided?

**Close session**

Thank you for your time today. We will develop a summary of the discussion and conclusions from today’s focus group and send out to you in the next few weeks.

Please do get in touch after the session if you think of any other interventions or comments you would like to make.
