# Supplementary material for: Co-designing interventions to ‘live well’: experiences and perceptions of the Genetic, Undiagnosed and Rare Disease (GUaRD) community
Source: J Community Genet. 2023 Mar 31;14(3):295–305. doi: 10.1007/s12687-023-00643-1 (PMC10063929; doi:10.1007/s12687-023-00643-1)
Supplement: Supplementary file 2 — Supplementary file2 (DOCX 16 KB) [file 12687_2023_643_MOESM2_ESM.docx]

**Workshop protocol**

**The Genetic, Undiagnosed and Rare Disease community: ‘Living well’**

**Welcome and establish ground rules for the focus group**

**Any questions before starting?**

**Overview to the research plan**

1. Share practical interventions that arose from the journals and focus groups in relation to day to day lives and engagement with the health system

2. Score the ideas using a framework via an app on your phone

**APEASE – explain the framework and scoring**

During the APEASE scoring example prompts include:

- *Affordability: Does this practical intervention require funding? Is there funding available?*
- *Practicability: Is it feasible to deliver?*
- *Effectiveness and cost-effectiveness: Does the benefit justify the cost?*
- *Acceptability: Do those the intervention affects consider it appropriate?*
- *Side-effects / Safety: Will the intervention have unwanted side-effects?*
- *Equity: Is the intervention fair?*

**Close session**

Thank you for your time today. We will develop a summary of the discussion and conclusions from today’s focus group and send out to you in the next few weeks.

Please do get in touch after the session if you think of any other interventions or comments you would like to make.
