# Supplementary material for: Co-designing interventions to ‘live well’: experiences and perceptions of the Genetic, Undiagnosed and Rare Disease (GUaRD) community
Source: J Community Genet. 2023 Mar 31;14(3):295–305. doi: 10.1007/s12687-023-00643-1 (PMC10063929; doi:10.1007/s12687-023-00643-1)
Supplement: Supplementary file 3 — Supplementary file3 (DOCX 22 KB) [file 12687_2023_643_MOESM3_ESM.docx]

**Supplementary file 3**. Detail of CoPICO analysis

Practical interventions identified by CoPICO (Context, Population, Intervention, Comparison and Outcome)

| **Contextual Topic** | **Population** | **Intervention** | **Comparison** | **Outcome** |
| --- | --- | --- | --- | --- |
| Social connection, activities, and hobbies with similar people (e.g., friends, GUaRD community)  “This is about social interaction and getting to know each other, joining in the conversations” (ID 1, Carer/Support sector)  “When your child has a rare syndrome, connecting with others who understand and know about the syndrome is important. Social media has made the world a lot smaller and we can easily find other families or individuals online to share stories and learn from each other. But face to face, real life interaction offers so much more” (ID 1, Carer/Support sector)  “I feel a level of isolation generally anyway, with not knowing anyone else with my condition or even what exactly it is” (ID 3, GUaRD)  “It’s so hard for my daughter to make friends when she has a chronic medical condition and spends a lot of time in hospital” (ID 27, GUaRD/Carer/Support sector) | GUaRD community | What: Arranging activities and social groups for the GUaRD community (and promoting balance)  Who: peer support groups | Little to no social connections | GUaRD: meet other people, improved connections, socialisation, enjoyment, independence, gain skills, alleviate sense of being ‘alone’    Carers: reduced workload, peace of mind    Program/organisation: spent time and resources |
| Accommodation for individuals with GUaRD in specialised activities  “We contact eight woodworking businesses in the Melbourne metro area and are pleased to find someone show an interest in helping [him] reach his goal.” (ID 1, Carer/Support sector)  “I do not cycle that much these days, as I have become too slow for my bike group.” (ID 9, GUaRD) | GUaRD community | Collect and compile a list of organisations, companies, or individuals who can accommodate individuals with GUaRD in specialised activities | Learning new skills or activities that stay within the limitations of an individual’s physical or intellectual capabilities | GUaRD: Escape the bounds of limitation, learn new skills, individual growth, maybe applicable to future jobs or studies |
| Career/education development  “Another parent had once said to me: ‘Just because they leave school, it does not mean they stop learning… they are learning the skills they need to be independent adults, whatever that might be for them.’” (ID 1, Carer/Support sector)  “I have zero faith in being able to find a job in the next 6 months. All of my experience is industrial or maritime, both fields I can no longer work in because of my dissociative episodes… This means any other job I am likely to qualify for is entry-level and I guarantee there is someone who has lost their job due to covid that is much more qualified than I” (ID 21, GUaRD)  “I have applied for my next masters and my hopefully-soon-to-be-supervisor thinks I can get in. I don’t, but I have a list of places I am going to apply to if I don’t get in” (ID 20, GUaRD/Support sector)  “Everyone keeps asking what I’m going to do now that school is over, and I have absolutely no idea but I’m sure I’ll figure it out soon” (ID 24, GUaRD) | GUaRD community | Arrange accessible education and work training opportunities.  Facilitated by training organisations (e.g., TAFE, hospitality courses etc.)    Utilise/encourage career guidance counselors | No employment; attempting to obtain and work an unsuitable job position | GUaRD: gain **independence**, develop skills, occupy time, feel a sense of purpose, gain employment  Carers: reduced workload, peace of mind  Training organisations: time and resources |
| Support facilitating independence for GUaRD children  “Part of this new independence is [my daughter] needed to take responsibility for her own health and medical appointments. This, like everything will be done very slowly and of course with much support.” (ID 2, Carer)  “This “adult transitioning” is wayyyy harder than I thought it would be. I stopped reminding her of many things… Then of course things went south rapidly with missed Tafe classes, appts and mess up with work start times etc etc etc.” (ID 2, Carer) | GUaRD community | Hosting workshops for GUaRD children to learn and better prepare for more independence and responsibilities | Allow children to learn as they go with little to no preparation depending upon the carer’s resources, time, and energy | GUaRD: gain independence, prepare ahead of time, alleviate some anxiety  Carers: Assistance with transitioning and teaching children, additional support as needed |
| Education supports  “In hindsight, perhaps I could have utilized the school’s student support centre but without any physical disability nor any kind of NDIS support I figured they would turn me away anyway.” (ID 21, GUaRD) | GUaRD community | More accessible support services for GUaRD students.    Facilitated by the education institution (e.g., University) | Not seeking/being reluctant to seek support. Not aware of which supports are available. | GUaRD: improved educational outcomes, feeling supported  Education institutions: time and resources |
| Online work/other commitments  “Moving forward, I hope that people have the option of conducting some of their work from home and that IT continues to be used to allow meeting between people in their own homes” (ID 9, GUaRD)  “Being able to access so many free activities all from the comfort of my home has made life so much easier for me as when I get tired I no longer have to worry about how I'm going to to get myself home.” (ID 23, GUaRD)  “This has been the first time in a long time I have felt included and been invited to online events which I could actually afford to attend” (ID 23, GUaRD) | GUaRD community | Working/studying/conducting other activities online (at home)    Facilitated by work/study/other activity organisers | Conduct activities face-to-face | GUaRD: more accessible and lower health risk  Organisations: time to coordinate, less people in work/activity environment |
| Accessible exercise  “In the virtual world, people of all different fitness levels and age groups come together, not only seniors or only people living with a chronic illness or injury and nobody seems to care if you need to take a break or you're doing something different to everyone else” (ID 23, GUaRD)  “I experience high levels of fatigue as a result of living with a rare disease and can only work part time which means I cannot afford the cost of a gym membership and so the only exercise I can get is the exercise which is free, mostly going for a walk. The problem is that all of the exercise groups that are specifically for people with chronic illness are unaffordable and inaccessible” (ID 21, GUaRD) | GUaRD community | Online fitness activities    Free/low-cost and tailored fitness activities | Not exercising | GUaRD community: improved fitness, physical and mental wellbeing  Fitness organisations: time and resources |
| Support for carers  “Since my first hospitalization in 2015… Mum has been there. Through all my recovery. For better or worse. I am certain she has reached a point of burnout. She snaps quickly and lies awake at night worrying about things… I wonder if she [my mum] blames herself? Either through her parenting… or because of my [condition]… Regardless, I don’t blame her in the slightest. I couldn’t have asked for better parents and it breaks my heart to think she blames herself for my misgivings.” (ID 21, GUaRD)  “It is so hard when the teachers ring and say “you” need to get her to be doing this and this and this. I can’t. They don’t live with her, they don’t see the at home with a brain injury.” (ID 2, Carer)  “Emotionally for myself I am feeling very drained and feeling very conflicted and guilty about the decisions I’m having to make for [my daughter] this year” (ID 29, Carer) | GUaRD carers | Psychological supports for carers of the GUaRD community    Facilitated by peer support groups, GPs, psychologists | Lack of support | GUaRD carers: more support, improved mental health and coping strategies, wider support system  GUaRD community: peace of mind that their carers are being looked after, alleviated feelings of guilt  Support group/services: time, resources, expertise |
| Database of rare disease specialists  “it is important to have access to a list of doctors who specialise in rare complex conditions, so we don’t waste money going to doctors who just don’t have the knowledge, experience and attitude required to work with complex rare patients” (ID 27, GUaRD/Carer/Support sector)  “This week I had 3 emails from people in our support group asking for support. I also had several questions on our fb page to be answered, asking for information and recommendations for doctors (ENT, opthamologists and gynaecologists). I was able to send them all information and doctors names from our list.” (ID 27, GUaRD/Carer/Support sector) | GUaRD community and carers | Compile a list/database of healthcare professionals who specialise/are experts in GUaRD conditions *(Note: I am not sure if it is feasible/good practice to list specific healthcare professionals. I have heard of support groups doing this informally – e.g., suggesting particular doctors/surgeons and hospitals)*  Facilitated by peer support groups | Receive treatment/management from whichever healthcare professionals is available or spend time searching for specialists | GUaRD community: receive improved care  GUaRD carers: peace of mind, less time taken to seek out particular specialists  Healthcare professionals: increased burden, patients might question the competence of healthcare professionals not listed on the database  Peer support groups: time and resources |
| Increased rare disease education for healthcare professionals (including referral pathways)  “I remember our GP saying [my daughter] should have the MMR booster. She had NO clue that “live” vaccines weren’t recommended to be given to ANE patients, when I questioned her she *thought* ANE was ONLY brought on by influenza” (ID 2, Carer)  “it doesn’t work for patients, especially when your doctors keep making mistakes or don’t keep up with new advances, or don’t want to learn about rare genetic conditions because they ‘won’t see another case for at least another 5 years’” (ID 27, GUaRD/Carer/Support sector)  “we had a new family in the States earlier in the year where the hospital printed off parts of our website and made sure the family knew of our support group.” (ID 2, Carer/Support sector) | Healthcare professionals | Compile information sheets on rare diseases to distribute to healthcare professionals    Facilitated by peer support groups | Not all healthcare professionals are up-to-date with GUaRD information | GUaRD community: better care  GUaRD carers: peace of mind  Healthcare professionals: better equipped, time  Peer support groups: time and resources |
| Accessible Telehealth    “While we have been making some progress in his transition to being an independent health care recipient, it has been disappointing to find that under telehealth, his independence has receded because his accessibility needs in this online setting have not been considered” (ID 1, Carer/Support sector)  “We all put in some money to get her an Apple Watch. We thought this would help her with her exercise regime at pilates, keeping track of her heart rate and tachycardia; it also has a falls alert (in case she has another [symptomatic attack]) and an emergency button; it even reminds her to stand up and move if she’s been sitting too long” (ID 27, GUaRD/Carer/Support sector) | Healthcare professionals/providers | Education on accessibility for healthcare professionals | Difficulty communicating, unproductive consultations with less face-to-face time for care | GUaRD: information on accessing telehealth, convenient appointments, less environmental/outside exposure (if immunocompromised)  Healthcare professionals: more knowledgeable in telehealth options, ability to meet with patients any time or anywhere |
| Mental health and wellbeing support for Individuals with GUaRD    “I need help, psychological help, I need to get back into doing all that, but its now gone from, ‘I’ll do that after this and that’ to ‘I can’t take any more steps in any (wrong) direction until I do this’” (ID 20, GUaRD)    “I think this style is something I need. Someone to keep me accountable for the behavioral issues and lack of discipline I exhibit in terms of my mental health” (ID 21, GUaRD)    “Nobody listens, nobody cares and I am forgotten” (ID 23, GUaRD)    “She [my therapist] seriously is so booked up with clients that next month was the soonest I could get in. This is why we need the Mental Health Care Plan to cover Telehealth sessions too. For rural and regional Australians who don’t have access to, or have such limited access to proper psychological help.” (ID 21, GUaRD) | GUaRD community | Provide mental health and wellbeing support (I.e. psychiatrist referrals, trained support group members to aid with coping, etc.) to individuals with GUaRD | Seeking and receiving mental health and wellbeing care would be left to the individual and their carer | GUaRD: improved mental health and wellbeing, learn coping mechanisms, regain sense of purpose and direction |
| Awareness of peer support    “Thank goodness for social media, we do weekly and sometimes daily searches and have had numerous families join us because we found them. Roughly translated this is what I found on Twitter. We think our group is easy to find but obviously some people do not find it so” (2, November) | GUaRD community | Share a list of support groups and their information throughout the GUaRD community for people to find them | No list of support groups and their information; leaves people to have to search far and wide for helpful connections | GUaRD: Connections to people with the same or similar conditions, builds community, access to relevant information regarding one’s condition(s)  Support groups: builds the community and platform, provides exposure to people to learn about the condition(s) |
| Linking with caseworkers/support workers    “We are back to the drawing board with support workers, our positive vibes and hope for success with the organisation we were working with has disappeared along with their lack of availability. With some advice from my brothers support coordinator we hope to be trialling another organisation very soon. The brain rehab helped in organising and it is amazing the results they can get that I can’t.” (ID 2, Carer/Support sector)    “We finally found a support worker that I think will be a great match for [my daughter]. [My daughter] really liked her and once it can be organised into a routine it should make everyone’s life easier” (ID 2, Carer/Support sector) | GUaRD community | Give advice or referrals to those looking for a caseworker or support workers | No sharing of information regarding helpful caseworkers and support workers | GUaRD: improved guidance and support for health or personal journey |
