# Supplementary material for: Co-designing interventions to ‘live well’: experiences and perceptions of the Genetic, Undiagnosed and Rare Disease (GUaRD) community
Source: J Community Genet. 2023 Mar 31;14(3):295–305. doi: 10.1007/s12687-023-00643-1 (PMC10063929; doi:10.1007/s12687-023-00643-1)
Supplement: Supplementary file 4 — Supplementary file4 (DOCX 4252 KB) [file 12687_2023_643_MOESM4_ESM.docx]

**On line resource 4**

APEASE framework results


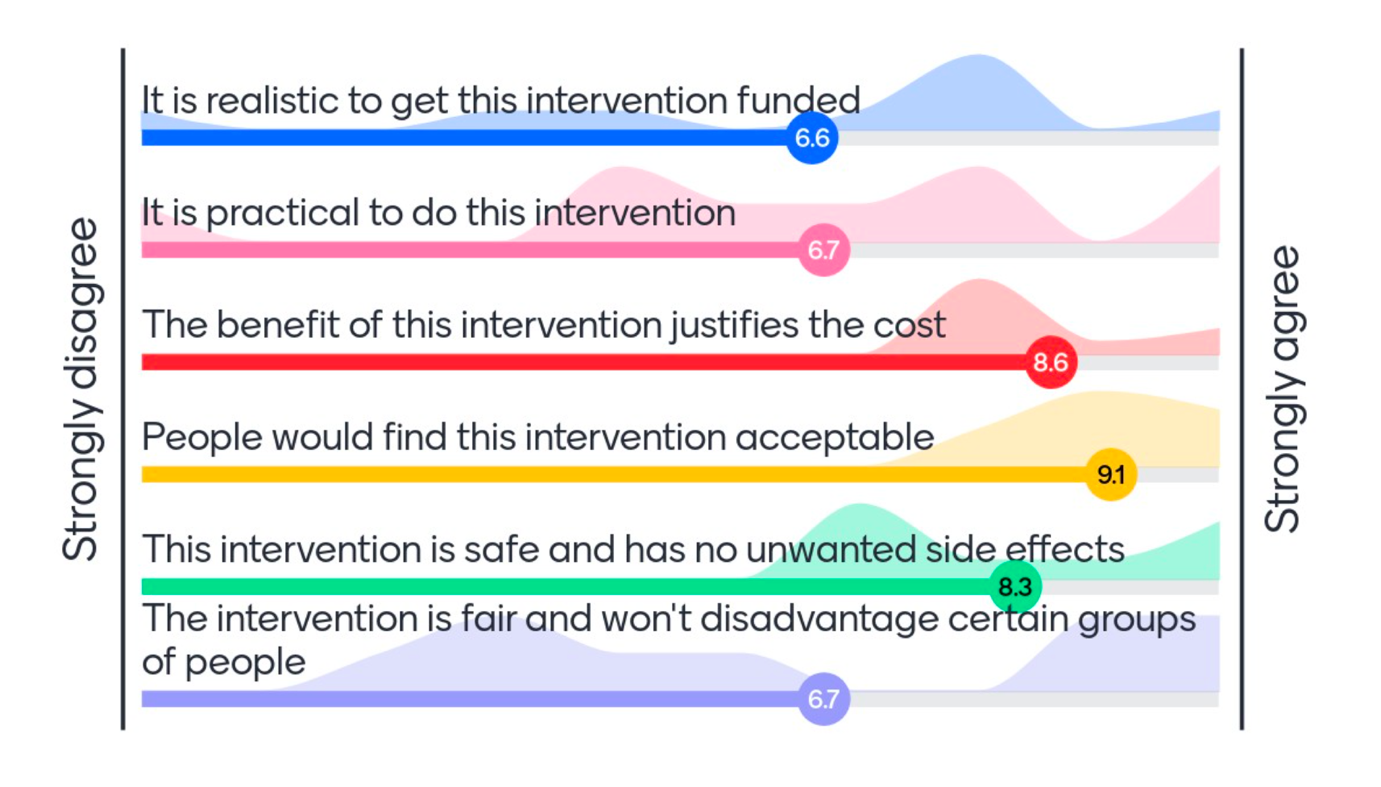


Figure 1 a Educating clinicians about peer support groups. Scale: 0 strongly disagree – 10 strongly agree


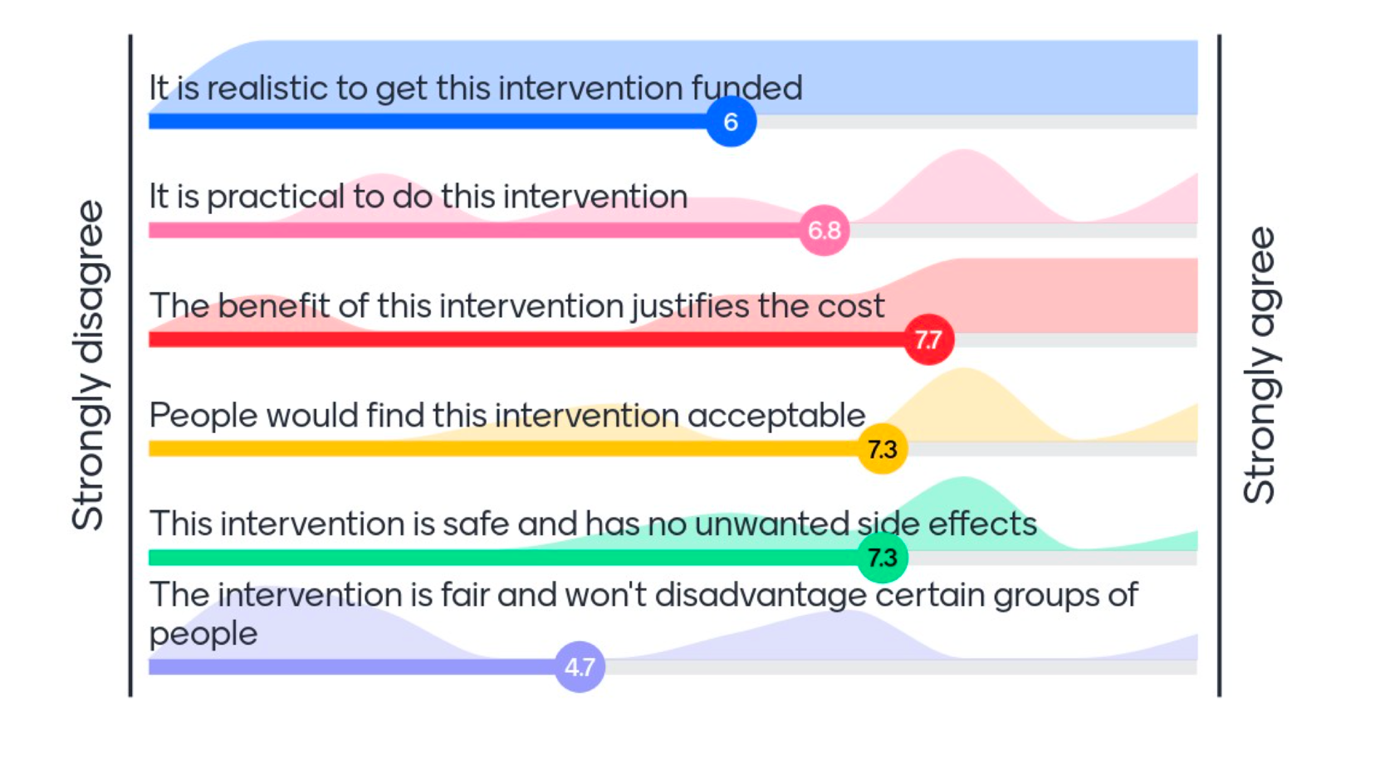


Figure 1 b Creating face-to-face catch ups in each state or within groups of people with GUaRD who share common traits Scale: 0 strongly disagree – 10 strongly agree


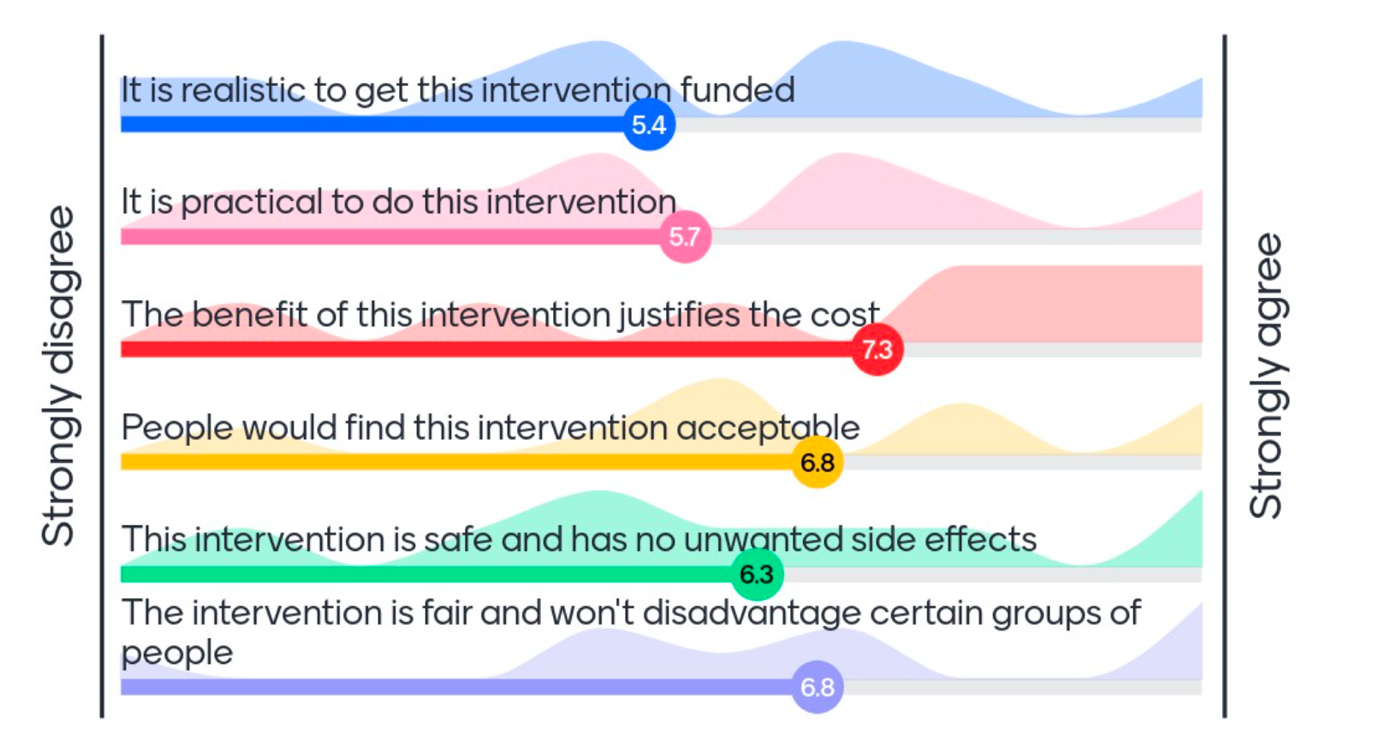


Figure 1 c Adding summaries of rare diseases to existing government or hospital registries for health professionals and consumers Scale: 0 strongly disagree – 10 strongly agree


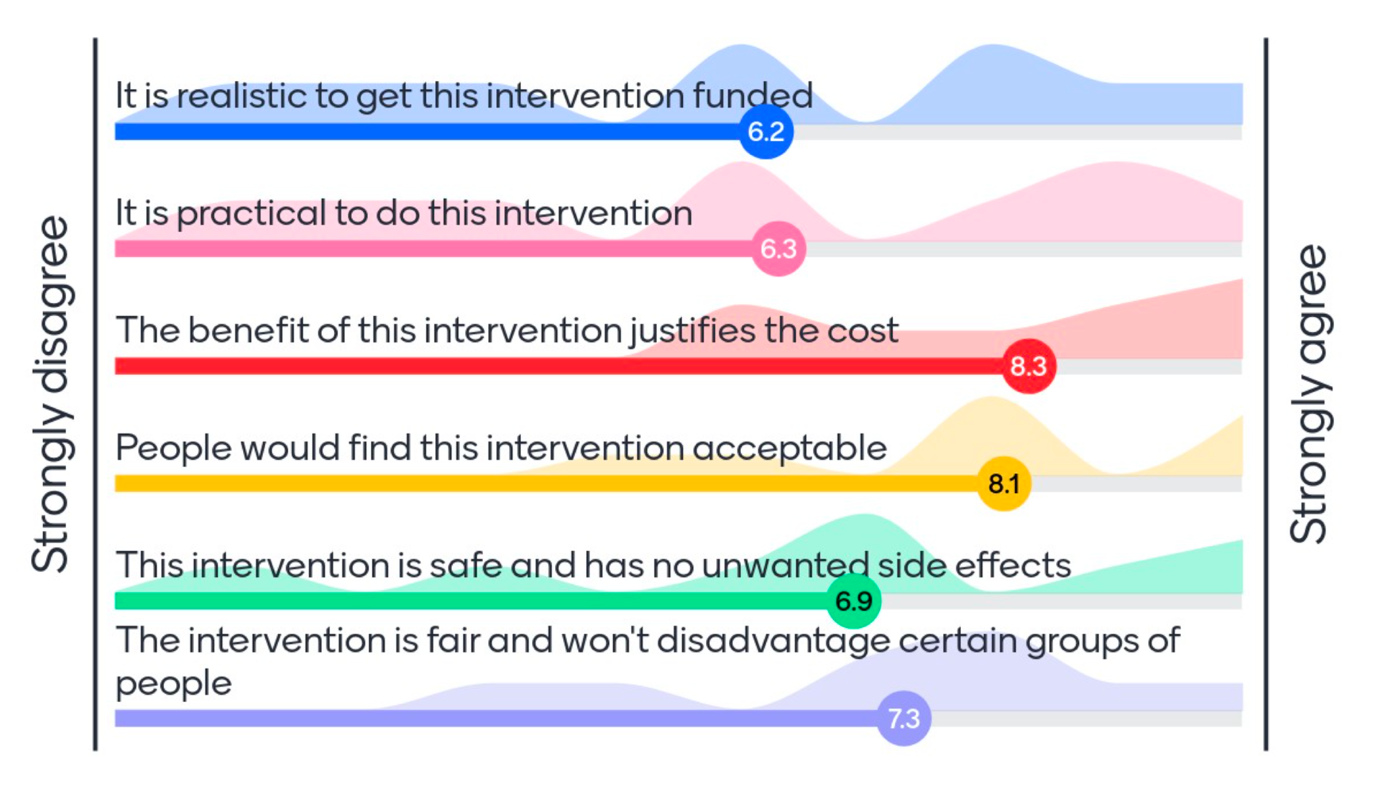


Figure 1 d Creating a database of mental health practitioners (e.g., psychologists) who are interested in rare disease and providing priority access to appointments Scale: 0 strongly disagree – 10 strongly agree


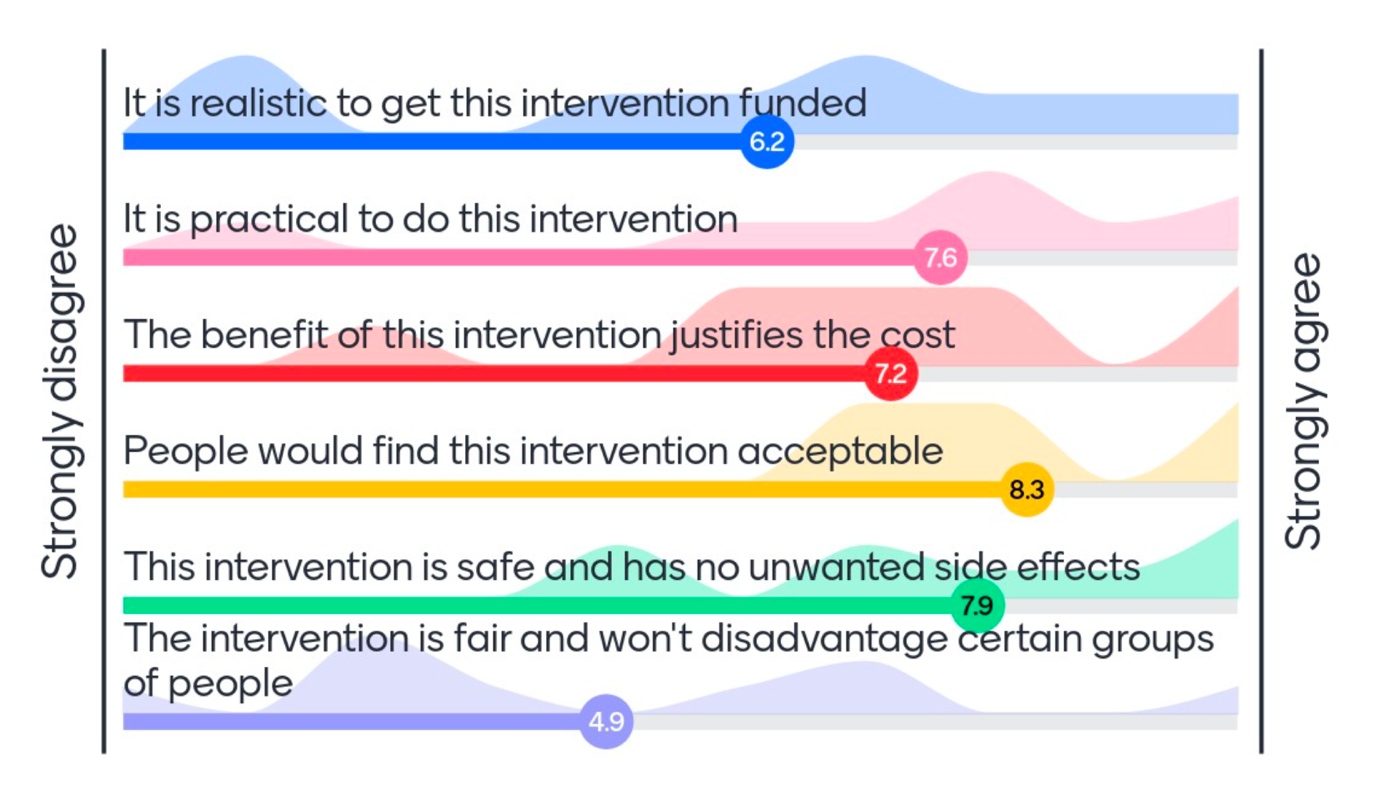


Figure 1 e Support in grant writing to access funds for peer support group resources Scale: 0 strongly disagree – 10 strongly agree


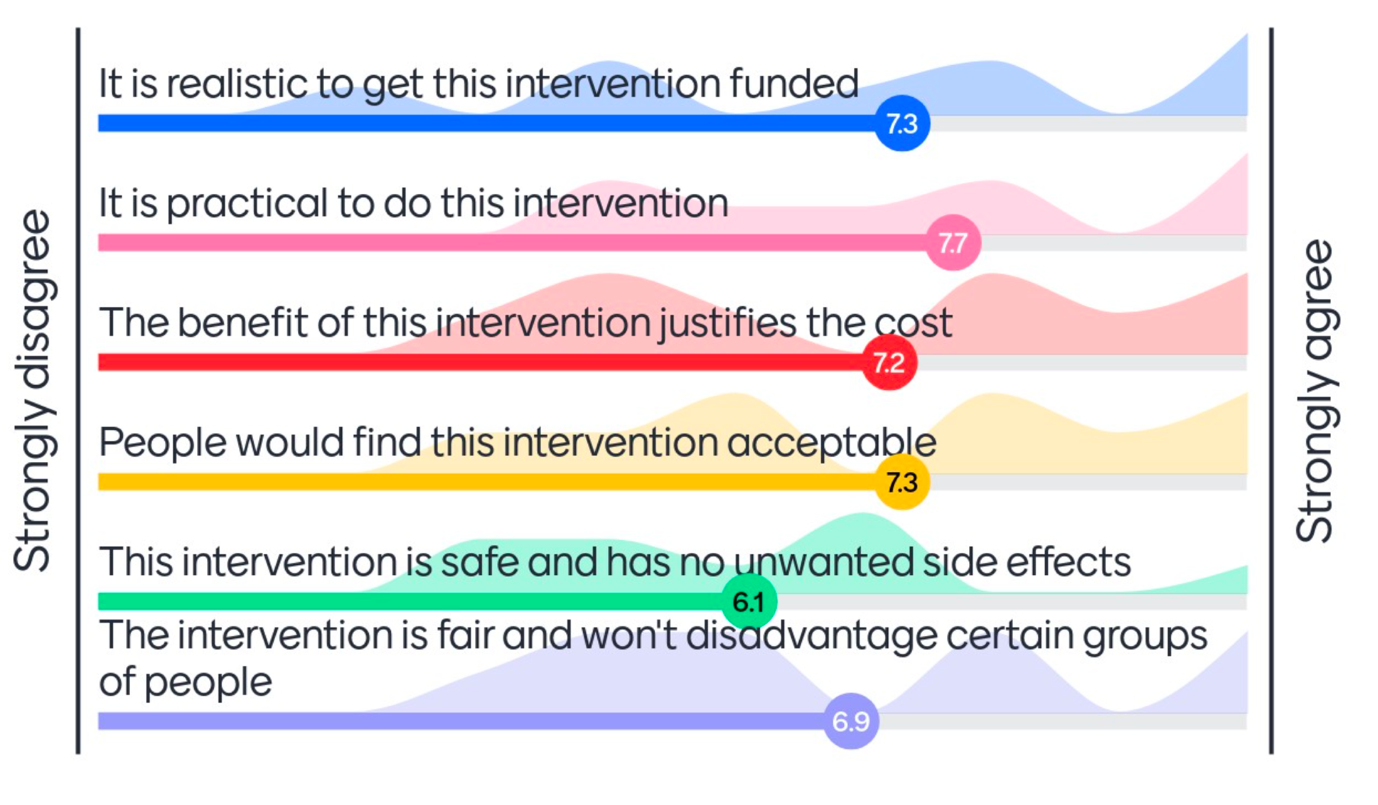


Figure 1 f A lived experience video library Scale: 0 strongly disagree – 10 strongly agree


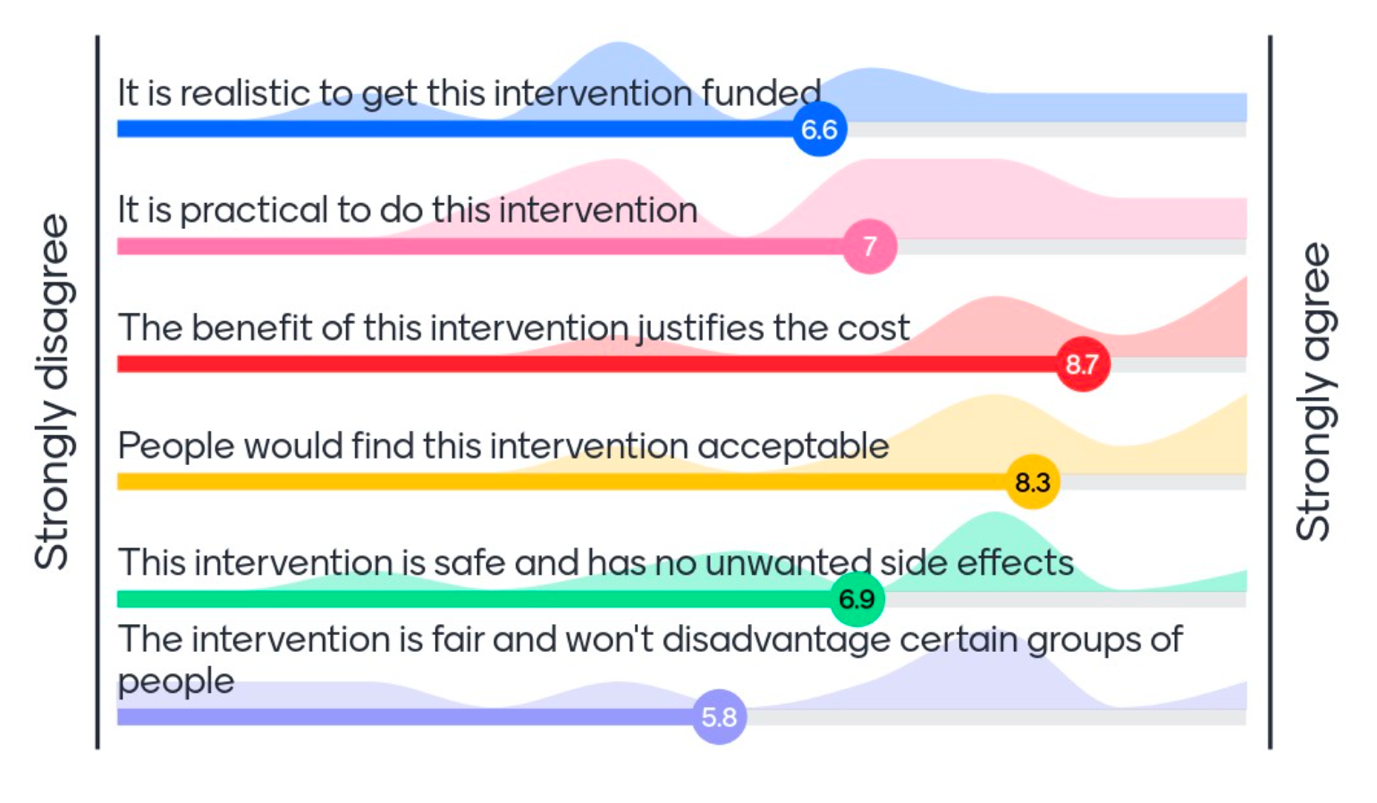


Figure 1 g Creating workshops that educate people with GUaRD and their families about transitioning into the workforce from school Scale: 0 strongly disagree – 10 strongly agree

Figure 1 h Connecting people with common traits Scale: 0 strongly disagree – 10 strongly agree
